# Comparison of the accuracy of immediate implant placement using static and dynamic computer-assisted implant system in the esthetic zone of the maxilla: a prospective study

**DOI:** 10.1186/s40729-022-00464-w

**Published:** 2022-12-13

**Authors:** Yuzhang Feng, Zhenya Su, Anchun Mo, Xingmei Yang

**Affiliations:** State Key Laboratory of Oral Diseases & National Clinical Research Center for Oral Diseases & Department of Oral Implantology, West China Hospital of Stomatology, Sichuan University, No.14, 3rd Section, Renmin Nan Road, Chengdu, Sichuan People’s Republic of China

**Keywords:** Accuracy, Computer-assisted surgery, Dental implant, Immediate implant placement, Dynamic navigation, Static template

## Abstract

**Purpose:**

This study aimed to compare the accuracy of fully guided between dynamic and static computer-assisted implant surgery (CAIS) systems for immediate implant placement in the esthetic zone.

**Methods:**

A total of 40 qualified patients requiring immediate implant placement in the esthetic zone were randomly and equally assigned to either static CAIS group (*n* = 20) or dynamic CAIS groups (*n* = 20). Global deviations at entry, apex, and angular deviation between placed and planned implant position were measured and compared as primary outcomes. Secondary outcomes were the deviation of implant placement at mesial–distal, labial–palatal, and coronal–apical directions.

**Results:**

For the immediate implant placement, the mean global entry deviations in static and dynamic CAIS groups were 0.99 ± 0.63 mm and 1.06 ± 0.55 mm (*p* = 0.659), while the mean global apex deviations were 1.50 ± 0.75 mm and 1.18 ± 0.53 mm (*p* = 0.231), respectively. The angular deviation in the static and dynamic CAIS group was 3.07 ± 2.18 degrees and 3.23 ± 1.67 degrees (*p* = 0.547). No significant differences were observed for the accuracy parameters of immediate implant placement between static and dynamic CAIS systems, except the deviation of the implant at entry in the labial–palatal direction in the dynamic CAIS group was significantly more labial than of the static CAIS (*p* = 0.005).

**Conclusions:**

This study demonstrated that clinically acceptable accuracy of immediate implant placement could be achieved using static and dynamic CAIS systems.

*Trial registration* ChiCTR, ChiCTR2200056321. Registered 3 February 2022, http://www.chictr.org.cn/showproj.aspx?proj=151348

## Introduction

A dental implant-supported restoration has widely been proven to be an adequate replacement for teeth loss with future long-term results [1]. Immediate implant placement has increasingly emphasized its advantages of shortening treatment time, less surgical trauma, maximally preserving the remaining bone and soft tissue, and optimizing esthetic success [2, 3]. Precision on the accuracy of the immediate implant placement in the esthetic zone is highly technically sensitive [4–6]. The existence of the irregular alveolar bone plate and the slope of the extraction socket interferes with proper visualization of the exact implant site [7]. The conventional freehand protocol makes it difficult to control the palatal bone’s resistance to ensure implant placement in the right location and orientation during the operation. Thus, these would result in compromising the accuracy of immediate implant placement. Poor positioning and angulation of the implant may lead to counterproductively functional and esthetical outcomes [8].

With the development of computer-assisted technology, static and dynamic CAIS techniques have been widely used to transfer the preoperative plan to the surgical site and achieve significantly high accuracy compared to the freehand protocol [9, 10]. In the static CAIS system, a surgical template is manufactured with an embedded metal sleeve tube guiding the location and orientation of the implant based on a virtual prosthetic-driven implant design. A fully guided static template provides guidance for preparing the borehole following the sequence of the drills and the implant insertion. The implant planning could not be modified during the surgery. The dynamic technique utilizes optical tracking technology for the real-time location of the surgical site and implant handpiece. The ideal implant position is presented on the preoperative CBCT data. The monitor screen demonstrates the deviations in the 3-dimensional section between the planned position and the actual drill location. Adjustments could be performed in real-time during the operation [11].

Dechawat Kaewsiri et al. reported that no statistical significance was found in implant deviation between two techniques in single tooth space, including anterior and posterior surgical sites, without limiting the timing of placement [12]. Jaemsuwan et al. demonstrated that in fully edentulous, no difference in the accuracy of implant placement was found between static and dynamic CAIS [13]. Po-Jan Kuo et al. presented in their study that the dynamic CAIS protocol could obtain optimal implant position and esthetical outcome in immediate implant placement [14]. However, there are scarce clinical studies that systematically compare the accuracy of dynamic and static CAIS applied for immediate implant placement in the esthetic zone. Thus, this study aimed to compare the accuracy of immediate implant placement in the esthetic zone using static and dynamic CAIS protocols.

## Materials and methods

### Experimental design

**Fig. 1:**
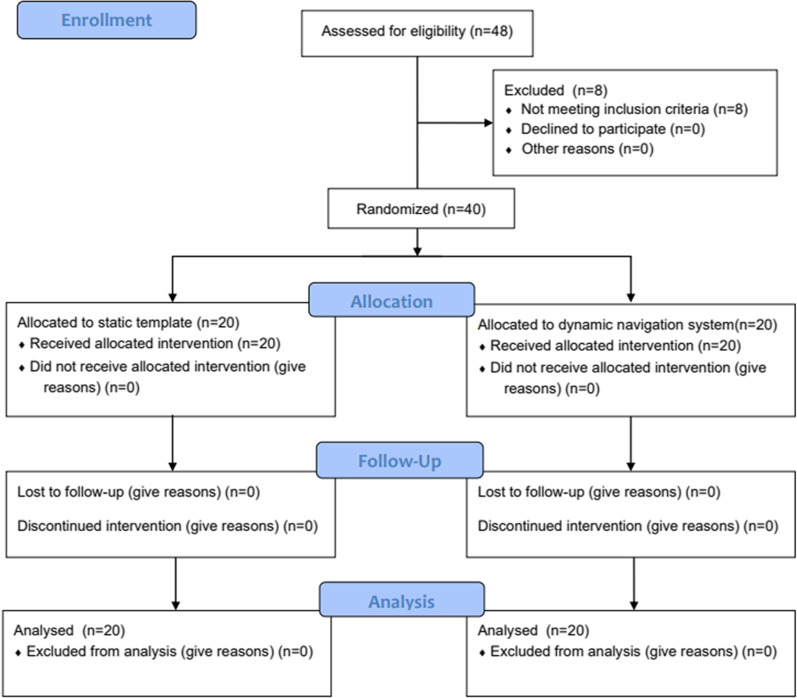
CONSORT flowchart

This study was a prospective randomized clinical trial to compare the discrepancy between the planned and actual implant positions in immediate implant placement following a static or dynamic CAIS. This study was conducted following the CONSORT statements. The CONSORT flowchart is shown in Fig. 1. The primary outcomes were the global deviation at the entry and apex points and the angular deviation between the planned and actual implant positions. The secondary outcomes assessed the implant placement deviation in mesial–distal, labial–palatal, and coronal–apical directions. This study was approved by the ethical committee of West China Hospital of Stomatology, Sichuan University (WCHSIRB-D-2021-540) and was registered in the Chinese Clinical Trial Registry database (ChiCTR2200056321). Informed consent from all patients enrolled was acquired following the principles of the Declaration of Helsinki. This study was conducted at the Department of Oral Implantology, West China Hospital of Stomatology.

### Patient selection

Between February 2022 and October 2022, 40 patients were recruited for this study who fulfilled the eligibility criteria. The inclusion criteria were as follows:

- An age of at least 18 years.
- The maxillary esthetic single tooth sites (13–23) could not be retained due to trauma, endodontic failure, root fracture, root resorption.
- Intact socket walls.
- The absence of acute infection at the site.
- Sufficient apical bone to allow implant placement with Insertion torque 25–40 Ncm [15].
- The length of the implant has at least 3–5 mm in contact with the alveolar bone.
- Good general health.
- Good oral hygiene status determined by plaque index < 10% [16].

The exclusion criteria were as follows:

- Defects of labial bone.
- Heavy smokers (over 10 cigarettes per day).
- Uncontrolled systemic diseases that may impair osseointegration or contradict implant surgery.

### Sample calculation

For the determination of sample size, the following calculation was based on the mean deviations at the implant platform between the static group and dynamic group reported in previous studies (0.73 ± 0.10 mm vs. 1.05 ± 0.44 mm) [12, 17]. The significance level (*α*) was set at 0.05, and the power (1 − *β*) was set at 0.80. The minimum required sample size resulted in 20 for each treatment group calculated by G*Power software (Version 3.1.9.7).

### Randomization

Blocked randomization was conducted using a computer-generated permuted block of four (IBM SPSS Statistics, 20.0; SPSS Inc., Chicago, USA). The allocation was concealed utilizing sealed opaque envelopes by an investigator not involved in the following surgeries and evaluations. Patients were randomly divided into static and dynamic CAIS groups. The blinding of participants and surgeons was not possible during the treatment delivery, while the outcome examiner was blinded to the grouping allocation.

### Preoperative virtual implant planning

The whole prosthetic-driven implant treatment was designed through a digital workflow. Preoperative cone beam computed tomography (CBCT) scan (J. Morita Inc., Kyoto, Japan) was performed at standardized settings (90 kV, 5 mA, 0.25 × 0.25 × 0.25 mm voxel size, 10 × 10 cm FOV) for every patient. For the dynamic group, a U-shaped registration device with radiopaque fiducial markers covering the surgical site was attached to the dentition during the scan procedure. The intraoral scan (3Shape, Copenhagen, Denmark) was performed, and the following prosthetic restoration was virtually designed by Exocad DentalCAD software (exocad GmbH, Darmstadt, Germany). The digital imaging and communication in medicine (DICOM) data from the CBCT scan were imported in implant planning software, either NobelClinician software (Nobel-Biocare, Kloten, Switzerland) for static CAIS group or dynamic navigation system (Digital-care, Suzhou Digital-health Care Ltd) for dynamic CAIS group. After segmentation and 3-dimension model construction of the surgical site matched with the prosthetic design stereolithography (STL) file, ideal implant positioning was planned based on the prosthetic-driven design concept and biological principles as follows:

- The mesial–distal dimension: The implant was at the midpoint of the mesial–distal restoration width and kept a safe distance of at least 2 mm from adjacent teeth.
- The labia–palatal dimension: The implant was placed in a palatal position, and a gap of at least 2 mm was preserved between the implant and the labial bone filled with a bone substitute.
- The coronal–apical dimension: The implant platform was at 3 mm apical to the bottom point of the ideal labial emergence. The implant screw hole was oriented towards the cingulum of the designed crown.

For the static CAIS group, the teeth-supported stereolithographic fully guided surgical template was manufactured (Surgical Guide UV, HeyGears Inc.). All preoperative implant planning was performed by one experienced surgeon (ZYS).

### Surgical protocol

**Fig. 2:**
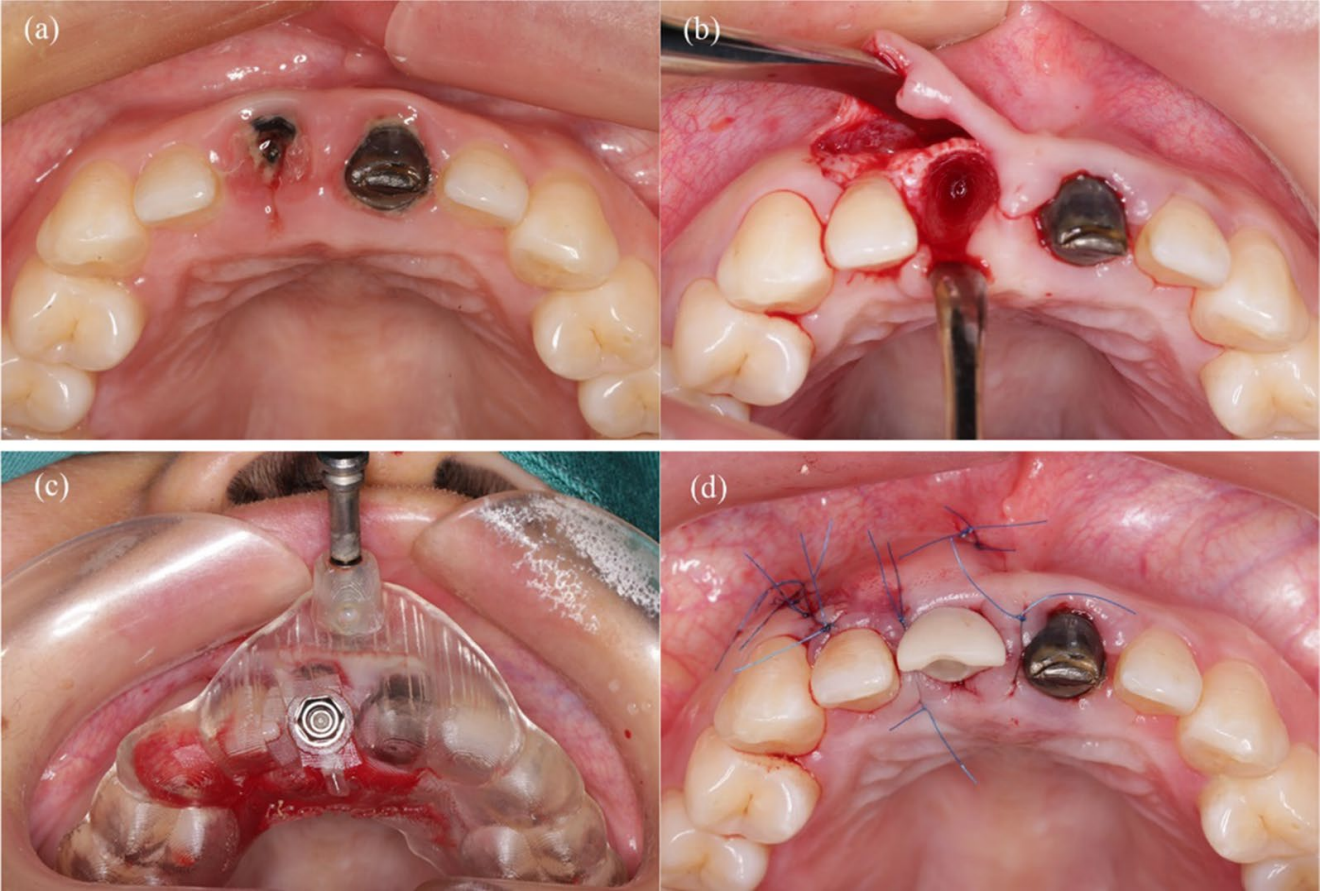
Surgical procedure for the static CAIS group. **a** Clinical observation of the hopeless tooth before surgery. **b** Extraction of 11. **c** Immediate implant placement with the fully guided static template. **d** Buccal gap was filled with bone substitute material and pre-fabricated temporary restoration was screwed

**Fig. 3:**
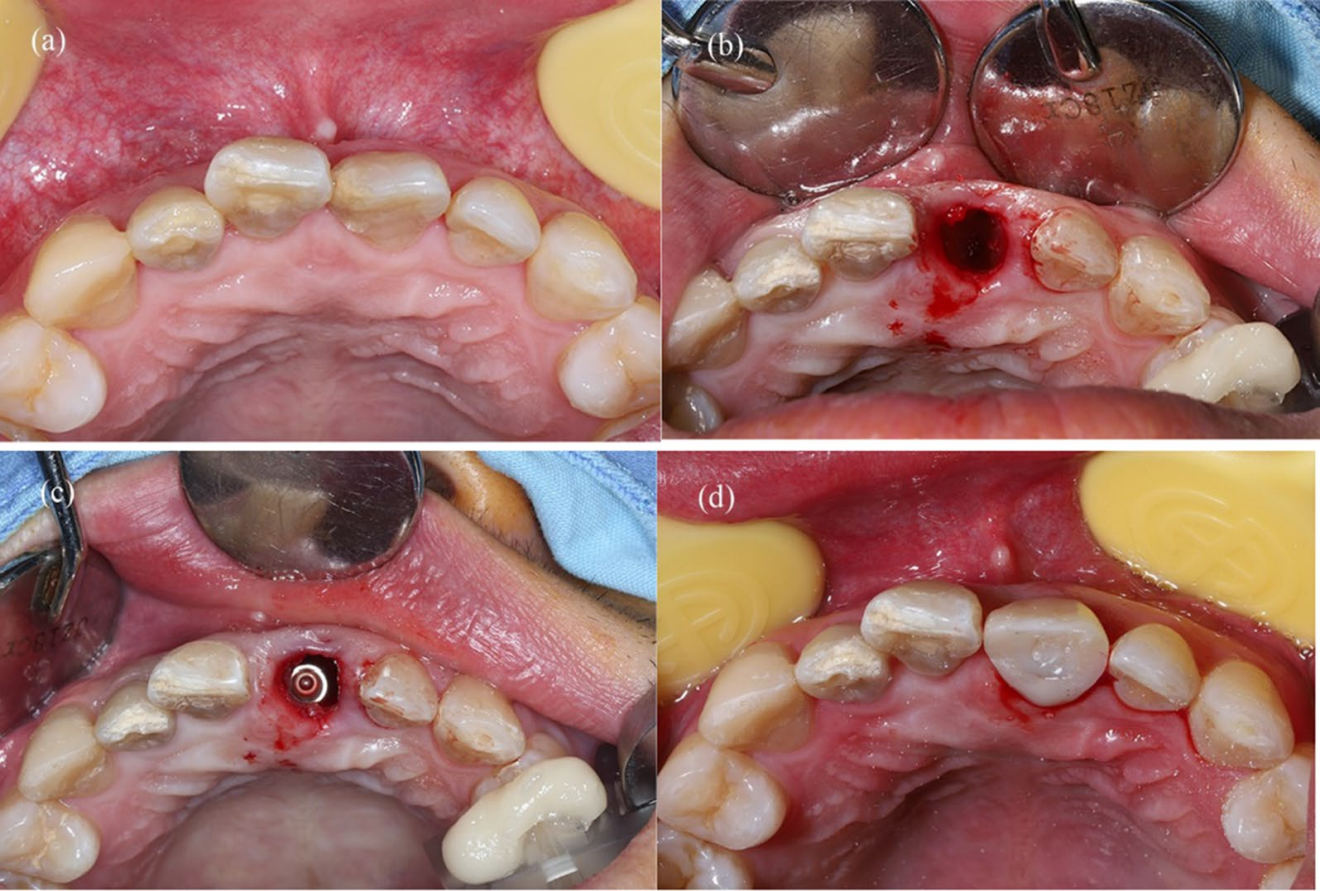
Surgical procedure for the dynamic CAIS group. **a** Clinical observation of the hopeless tooth before surgery. **b** Extraction of 11. **c** Immediate implant placement under the fully guidance of the dynamic CAIS system. **d** Buccal gap was filled with bone substitute material and pre-fabricated temporary restoration was screwed

All surgeries were performed by an experienced implant surgeon (XMY) who was familiar with both static and dynamic CAIS systems. The surgical procedures are presented in Figs. 2 and 3. After minimally invasive tooth extraction, each alveolar socket was thoroughly debrided and rinsed. The osteotomy preparation and tapered-implant insertion were performed according to the standardized fully guided protocol for each group (NobelActive, Nobel Biocare, Goteborg, Sweden; Bone Level Tapered, Straumann, Waldenburg, Switzerland). The osteotomies were prepared based on the manufacturer standard protocol. Once the insertion torque reached 35 Ncm or more, a pre-fabricated provisional crown was screwed to the implant using a temporary abutment. Submerged healing was applied if the primary stability failed to meet the requirement. The buccal gap was filled with the bone substitute material (Bio-oss, Geistlich, Inc., Bern, Switzerland). The postoperative CBCT was scanned with the same settings as the preoperative one.

### Static CAIS group

Upon verifying the correctly intraoral position, the anchor pin was inserted to confirm the fit and stability of the surgical template in situ. The osteotomy preparation was based on the drill sequence following the fully guided surgery protocol. For each drill, the corresponding drill stop was related to the depth of the borehole. After the preparation of the borehole, the implant was installed with the complete guidance of the surgical template in situ. During the operation, copious irrigation and an “in-an-out” drilling motion were inevitably required to avoid overheating.

### Dynamic CAIS group

Prior to the surgery, calibration was performed by mapping the calibration long and short ball drills to the body of the reference frame to determine the relationship between the geometry of the surgical site and the axis of the drill. The reference frame was retained to the dentition by flowable bis-acryl composite resin (Cool Temp Natural, Coltène). Then registration procedure was performed by locating the short drill tip to the fiducial markers attached to the U-shaped template to provide a link between the preoperative planning coordinate system and a real-time intraoperative coordinate system. An infrared tracking camera was set to detect the movement of the handpiece and the patient. Once completed, the U-shaped template was removed. Preparation of the borehole and the insertion of the implant were conducted under the guidance of the dynamic navigation system (Dcarer, Suzhou, China). A calibration of every drill before motoring was performed by positioning the tip of the drill to the cusp of the adjacent teeth. The surgeon examined the position of drills oriented in accordance with the 3D images on the monitor.

### Deviation measurement

**Fig. 4:**
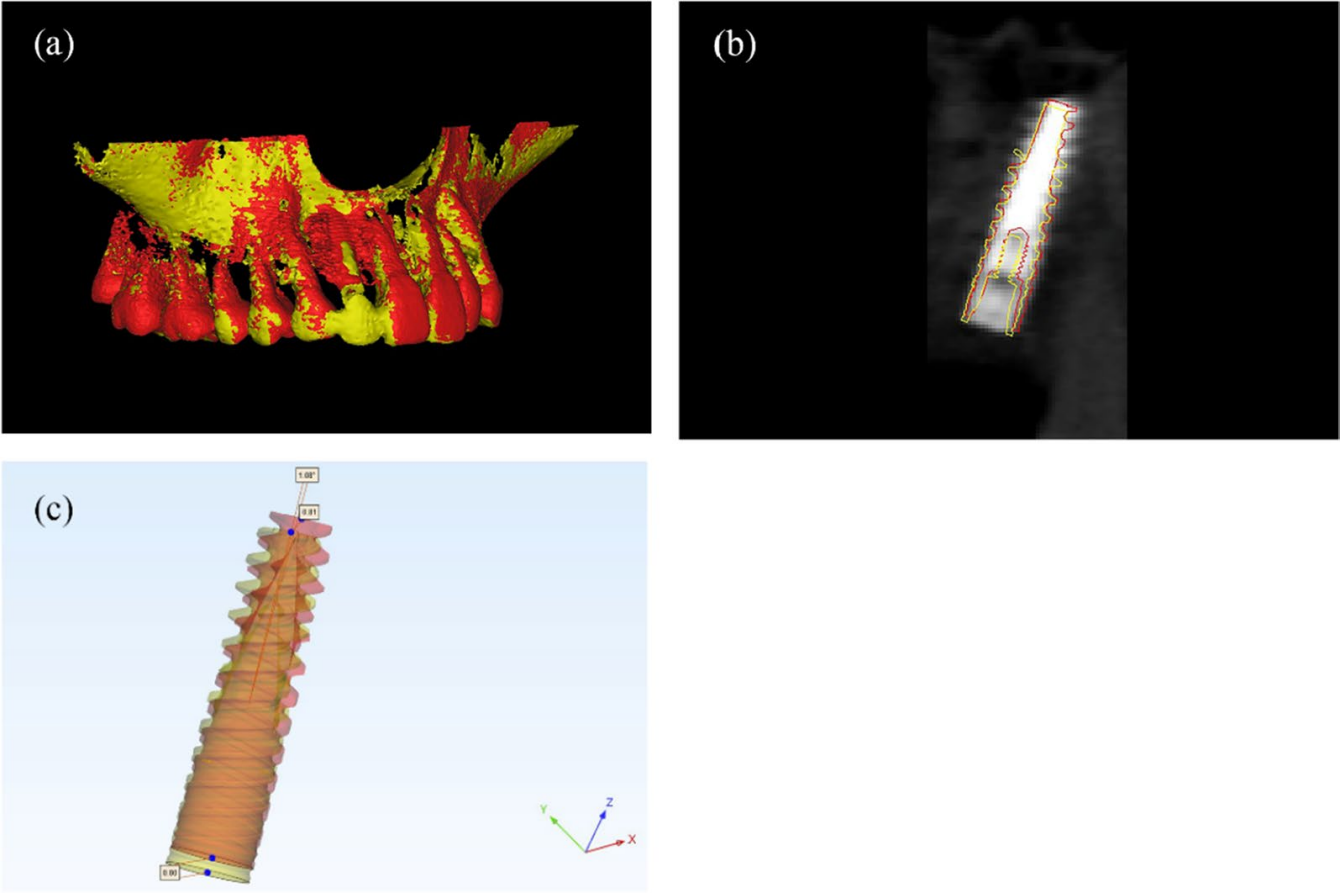
Measurement procedure. **a** Registration of the preoperative plan in STL format (colored yellow) and postoperative CBCT (colored red). **b** Registration of the preoperative implant plan (outlined in yellow) and postoperative implant position (outlined in red). **c** Measuring angular deviation, coronal, and apical global deviation of planned (colored yellow) and placed (colored red) implant position

The postoperative assessment was performed by one examiner (YZF). The preoperative plan was imported into Mimics software (Materialise NV 2018, Version 21.0). The planned implant position was determined, and a mask of the maxilla was created. A three-dimensional reconstruction calculated from the bone mask was exported as an STL file (colored in yellow). These steps were repeated to create the bone mask and the placed implant from the postoperative CBCT scan (colored in red). Therefore, a link between the two coordinate systems of two CBCT scans was established by matching residual dentition and bone anatomical landmarks throughout surface registration. The space relationship between the planned and placed implants was determined through the maxilla bone in the preoperative and postoperative CBCT data. Then, a mask of the placed implant was created in the postoperative data. The contour of the implant was derived from the mask and registered to the corresponding typical implant engineering STL document for measurement (outlined in red). Afterwards, following the bone data, the planned implant position (colored in yellow) and the placed implant position (colored in red) were exported as a separate STL file, respectively, and imported into 3-matic Medical software (Materialise NV 2018, Version 13.0) for deviation measurement (Fig. 4).

**Fig. 5:**
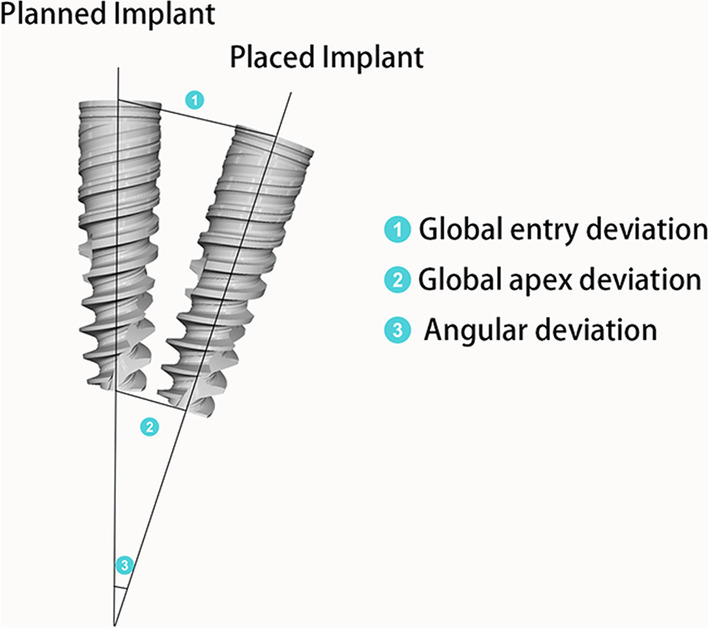
Accuracy measurement of the primary outcomes

Deviations between corresponding planned and placed implants were measured. The primary outcomes were according to the following parameters (Fig. 5):

- Global entry deviation: The linear 3-dimensional displacement between planned and actual implants, measuring at the center point of the implant platform.
- Global apex deviation: The linear 3-dimensional displacement between planned and actual implants, measuring at the center point of the implant apex.
- Angular deviation: The deviation between the central axis of the planned and actual implants.

The secondary outcomes assessed the implant placement deviation in mesial–distal, labial–palatal, and coronal–apical directions. The measurement results could be analyzed for the vector of deviations.

### Statistical analysis

One independent investigator conducted the data analysis using the Statistical software program (IBM SPSS Statistics, 20.0; SPSS Inc., Chicago, USA). The categorical variables were expressed in frequency and percentage and analyzed by Chi-square test to test group differences. The continuous data were presented as means ± standard deviations (SD). After verifying the normality assumption with the Shapiro–Wilk test and assessing the equality of variance with the *F* test, the Student’s two sample *t*-tests were used to determine the differences when data met normality and variance homogeneity requirements. When the distribution was not normal, the Mann–Whitney *U* tests were performed. The significance level *α* was set at *p* < 0.05.

## Results

A total of 40 patients (9 male patients and 11 female patients in the static CAIS group, nine male patients and 11 male patients in the dynamic CAIS group) were included in this study and randomly allocated into two groups. Each qualified patient’s gender, age, implant site, surgical protocol, and bone density are presented in Table 1. The implant survival rate was 100% in both groups. Table 2 presents the primary outcomes of the global entry, apex, and angle deviations between the planned and placed implant position. No significant differences in accuracy were found between static and dynamic CAIS groups. The deviations in mesial–distal, labial–palatal, and coronal–apical directions were measured and compared in terms of two groups (Table 3). Statistically, significance was found in the entry deviation at the labial–palatal plane between the two groups (*p* = 0.005).

**Table 1:** Demographics and surgical data of involved patients

Group	Static (*n* = 20)	Dynamic (*n* = 20)	*p*-value
Mean age (year)	42.60 ± 12.83	36.40 ± 13.11	0.139
Gender (*n*)			1.000
Male	9 (45%)	9 (45%)	
Female	11 (55%)	11 (55%)	
Implant position (*n*)			0.429
Central incisor	16 (80%)	13 (65%)	
Lateral incisor	4 (20%)	7 (35%)	
Surgical protocol (*n*)			0.429
Flap	15 (75%)	12 (60%)	
Flapless	5 (25%)	8 (40%)	
Bone density (HU)	596.01 ± 24.20	568.73 ± 30.56	0.488
Implant system			0.261
BLT Straumann	0	16	
NobelActive	20	4

**Table 2:** The primary outcomes of global implant deviations at entry, apex, and angular deviation (mean ± SD)

Group	Static (*n* = 20)	Dynamic (*n* = 20)	*p*-value
Global entry deviation (mm)	0.99 ± 0.63	1.06 ± 0.55	0.659
Global apex deviation (mm)	1.50 ± 0.75	1.18 ± 0.53	0.231
Angular deviation (°)	3.07 ± 2.18	3.23 ± 1.67	0.547

**Table 3:** The secondary outcomes of deviations at mesial–distal, labial–palatal, and coronal–apical directions (mean ± SD)

Group	Static (*n* = 20)	Dynamic (*n* = 20)	*p*-value
Entry deviation^a^ (mm)			
Mesial–distal	− 0.26 ± 0.43	− 0.16 ± 0.34	0.425
Labial–palatal	0.01 ± 0.58	0.53 ± 0.53	0.005*
Coronal–apical	0.44 ± 0.81	0.59 ± 0.78	0.556
Apex deviation^a^ (mm)			
Mesial–distal	− 0.51 ± 0.86	− 0.26 ± 0.85	0.346
Labial–palatal	− 0.01 ± 0.95	0.34 ± 0.50	0.151
Coronal–apical	0.50 ± 0.81	0.63 ± 0.75	0.618

^a^A positive at mesial–distal deviation represented that the actual implant position was placed more mesial than the planned one, and vice versa. A positive at labial–palatal deviation represented that the actual implant position was placed more labial than the planned one, and vice versa. A positive at coronal–apical deviation represented that the actual implant position was placed deeper than the planned one and vice versa

^*^p < 0.05

## Discussion

Accurate transferring of the preoperative implant plan to the surgical site is essential for appropriate restoration to ensure functional and esthetic outcomes, especially for immediate implant placement in the esthetic zone [4–6]. CAIS system nowadays typically contains static and dynamic technological pathways [18]. Clinical evidence has proved that both systems have currently achieved significantly higher accuracy of implant placement than the freehand protocol [6, 7, 13, 19].

In this study, analyzed as primary outcomes, the average global deviation at entry and apex in the static CAIS group was 0.99 ± 0.63 mm and 1.50 ± 0.75 mm, while that in the dynamic CAIS group was 1.06 ± 0.55 mm and 1.18 ± 0.53 mm. The angular deviation in the static and dynamic CAIS groups was 3.07 ± 2.18 degrees and 3.23 ± 1.67 degrees, respectively. Ali Tahmaseb et al. reported that the meta-analysis for the accuracy of the static CAIS in 20 clinical trials revealed a global deviation of 1.2 mm at entry, 1.4 mm at apex, and an angular deviation of 3.5° [20]. Adrià Jorba-García et al. analyzed that the mean global deviation for the dynamic CAIS system was 1.03 mm at entry and 1.34 mm at apex, as well as an angular deviation of 3.68° in clinical studies [21]. The present results are consistent with several systematic reviews and meta-analyses and have achieved the acceptable accuracy level of clinical requirement. In this study, no significant differences were found in implant deviation parameters between static and dynamic CAIS groups (*p* > 0.05), except for the vector deviation in the labial–palatal direction (*p* = 0.005). The accuracy was 0.01 ± 0.58 mm in the static group and 0.53 ± 0.53 mm in the dynamic group. The position of the implant platform in the dynamic CAIS group was more labial than that in the static CAIS group. A possible explanation for this observation could be the restriction of the guide sleeve from the static template during the preparation of the borehole and implant placement, which may partially offset the resistance of the palatal bone wall of the extraction socket during immediate implant placement [22]. Robert Stünkel et al. reported in their in vitro study that the dynamic CAIS system could achieve higher accuracy than the pilot drilling guide technique in posterior mandibular region of plastic models [23]. Zhaozhao Chen et al. compared the deviation of the static CAIS and conventional freehand protocol in human head cadavers for immediate implant placement at the maxillary incisor site [4]. In the guided group, the average angular deviation was 3.11°, as well as global deviation at entry and apex was 0.85 mm and 0.93 mm, respectively. Miaozhen Wang et al. compared the accuracy of implant placement between two CAIS systems in study models simulated post-extraction sockets [24]. The average global deviation at entry, apex, and angular deviation was 1.24 mm, 1.69 mm, and 3.44° in the static CAIS group and 0.60 mm, 0.78 mm, and 2.47° in the dynamic CIAS group, respectively. However, these in vitro studies were conducted in cadavers providing better visual field and operation access and being absent for intraoral patient conditions. Shimin Wei et al. reported that the global deviation at entry and apex was 1.01 mm and 0.88 mm in the immediate implant placement using dynamic CAIS protocol [7]. However, there were only 12 patients involved.

Generally, two types of the CAIS technique have been introduced in the previous literature, described as static and dynamic guidance systems [25]. The static approach utilizes a pre-manufactured surgical template to guide drills and implants sequentially to the ideal planned position. No intraoral modifications are allowed to make once the surgical template is in position. This protocol, therefore, is referred to as static guidance. It has been reported that full guidance could significantly increase the accuracy of the static CAIS, especially at fresh extraction sockets [26]. On the contrary, the dynamic approach does not rely on the guidance of any physical instrument. Dynamic navigation system utilizes optical tracking equipment to detect the movement of sensors attached to the implant handpiece and the surgical site. The 3-dimensional deviation between the ideal implant position and the actual drill location is illustrated on the monitor. If necessary, implant size and position changes could be made intraoperatively.

Acceptable accuracy of the immediate implant placement using CAIS systems depends on several factors during the clinical treatment, such as imaging factor, application factor, and human factor [27]. For imaging error, the layer thickness and voxel sizes contribute to the accuracy [28]. Besides, the preoperative and postoperative CBCT need to maintain the same parameters for the deviation measurement. A systematic review reported that the fracture of the template was one of the most common intraoperative complications [10]. There was no fracture of the guide or sleeve disintegration from the template in this study. For applying the static surgical template, the surgeon firstly positioned the drill into the sleeve before activating the motor. Van Assche et al. reported a noticeable tolerance caused by the gap between the drill and the interface of the metal sleeve, which results in rotation of the drilling from the right direction [29]. Clinically the surgeon needs to position the drill parallel to the inter wall of the sleeve to avoid unwanted lateral deviation [30, 31]. Besides, it is reported that this intrinsic error could be controllable in the fabricate phase [32, 33]. For the application of the dynamic navigation system, the calibration and registration procedures are essential for the achievement of higher accuracy. Though the drill and surgical site’s motion are tracked in real-time, the CBCT images are undertaken with a registration device before the surgery. Therefore, the calibration must be performed before drilling with every change of drills to ensure accuracy in implant drilling and placement [25]. Compared to a static system, a dynamic navigation system has an inherently reasonable learning curve to allow for proficiency to be achieved [34]. In this study, the surgeries were performed by a well-experienced surgeon to avoid the effect of the learning curve.

The present study still shows limitations. The comparative data of long-term clinical and esthetic benefits of implant placement by two CAIS techniques should be fully recorded. Besides, nowadays, patient-centered health care comes into the spotlight to empower patients to become active participants in their care. Therefore, patient-centered judgment should be evaluated further.

## Conclusion

In this study, the static and dynamic CAIS systems could achieve similar accuracy for the immediate implant placement in the esthetic zone. Further studies are essential for evaluating the benefits in terms of esthetical and patient-centered outcomes.

## Data Availability

All data generated or analyzed during this study are included in this published article.
